# Prevalence and risk factors of functional gastrointestinal disorders in Vietnamese infants and young children

**DOI:** 10.1186/s12887-022-03378-z

**Published:** 2022-05-27

**Authors:** Loo Wee Chia, Thi Viet Ha Nguyen, Van Nha Phan, Thi Thao Nguyen Luu, Gia Khanh Nguyen, Serene Yaling Tan, Shaman Rajindrajith, Marc Alexander Benninga

**Affiliations:** 1 FrieslandCampina, Amersfoort, The Netherlands; 2 FrieslandCampina Development Centre AMEA,, 89 Science Park Drive, #02-05 The Rutherford B, Science Park 1, Singapore, 118261 Singapore; 3 Department of Paediatrics, Hanoi Medical University, Hanoi, Vietnam; 4 National Paediatric Association Vietnam, Hanoi, Vietnam; 5 Department of Paediatrics, Faculty of Medicine, University of Colombo, Colombo, Sri Lanka; 6 Department of Paediatric Gastroenterology, Emma Children’s Hospital, Amsterdam University Medical Centres, Amsterdam, 1105 AZ The Netherlands

**Keywords:** Colic, Constipation, Diarrhoea, Dyschezia, Paediatric, Regurgitation, Rome IV, Toddler

## Abstract

**Background:**

Functional gastrointestinal disorders (FGIDs) are common early in life. The prevalence of FGIDs varies among countries but is unknown in Vietnam. The aim of this study is to assess the prevalence of FGIDs in healthy Vietnamese infants and young children.

**Methods:**

This was a cross-sectional, observational study involving healthy infants and young children between 0 – 48 months old in Hanoi, Vietnam. A representative total of 1511 subjects completed the validated questionnaire on paediatric FGIDs. Rome IV criteria were used to define FGIDs.

**Results:**

For Vietnamese infants (0–6 months) and young children (7–48 months), the prevalence of having at least one FGID was 10.0% and only 0.6% was having more than one FGID. Infantile regurgitation (9.3%) was the most prevalent FGID among infants 0—6 months of age while all other FGIDs had a low prevalence between 0—2.5%. For young children between 7 – 48 months old, functional constipation was the most common disorder at the rate of 5.6%. Association analysis revealed that the risk of infant regurgitation was significantly lower among infants with exclusively breastfeeding at 2 – 3 months and 3 – 4 months, formula initiation at 0 – 1 months, and higher paternal education level. The prevalence of functional constipation was significantly higher in male subjects, children in families with annual household income between 273,000,000 – 546,999,999 VND (or estimate around 11,800 – 23,800 USD), families with one child only, and formula feeding initiation at 1 – 2 months.

**Conclusions:**

The prevalence of FGIDs in Vietnamese infants and young children is relatively low compared to the published literature using Rome IV diagnostic criteria. Infantile regurgitation was the most common FGID in Vietnamese infants while functional constipation was most prevalent among Vietnamese young children.

**Trial registration:**

Netherlands Trial Registry Identifier: NL7286/NTR7495.

**Supplementary Information:**

The online version contains supplementary material available at 10.1186/s12887-022-03378-z.

## Background

Functional gastrointestinal disorders (FGIDs) are characterised by chronic or recurrent gastrointestinal symptoms with no evident organic aetiology related to structural or biochemical abnormalities [1]. FGIDs are common worldwide and the prevalence may vary with cultures, ethnicities and geographical areas [2]. The diagnosis of FGIDs relies on the symptoms-based Rome criteria [3]. Globally, an estimated 27 – 38% of infants and young children meet the criteria for at least one FGID, and over 20% present with two or more disorders [4].

FGIDs adversely impact the quality of life for both the children and their caregivers [1, 5–7]. Particularly, FGIDs during infancy increases the vulnerability for postpartum depression in mothers, poor parental-infant bonding and sub-optimal achievement of developmental milestones in infants and young children [8, 9]. Furthermore, it poses great burden to the families and the healthcare system as gastrointestinal disorders are the major reasons that prompt caregivers to seek medical advices [4]. The management of FGIDs emphasises on providing parental reassurance and nutritional advices [1, 10, 11] although further diagnosis and medication prescription are often adopted to address parental demands [12].

Demographically, over 50% of the world’s children population resides in Asia [13]. However, the epidemiological data about paediatric FGIDs in Asia is limited with no available data in Vietnam [14–17]. This study aims to investigate the prevalence and risks factors of FGIDs in Vietnamese infants and young children using ROME IV diagnostic criteria [1].

## Methods

### Study design and geographic details

We performed a cross-sectional study to assess the community prevalence of FGIDs in infants and toddlers, aged 0–48 months in Vietnam. Recruitment was conducted in two study sites in Hanoi, including a governmental hospital and a governmental kindergarten. Subjects aged 0 – 6 months were recruited from the vaccination centre in the National children’s Hospital while subjects aged 6.1 months – 48 months were recruited from both the vaccination centre as well as the kindergarten. Subjects were screened by paediatricians and researchers for eligibility and then recruited on the same day. Healthy infants and young children were recruited with the exclusion for subjects with physician-identified organic disorders, physical and/or mental deficiency, history of gastrointestinal surgery, and the use of long-term medication.

### Informed consent and data collection

Data was collected from October 2018 to March 2019. Written informed consent was obtained from the parent/legal guardian after providing details of the survey and all concerns answered, before the questionnaire was administered. A previously validated questionnaire to diagnose FGIDs in infants and young children was used for data collection [18–20]. The questionnaire was translated into Vietnamese language by a professional translation company and tested for clarity and applicability using a smaller sample of mothers of infants and young children. The final version of the questionnaire (in English language) is provided as a supplementary file (see Supplementary file 1). The questionnaire for FGIDs was tailored for the group of infants aged 0 – 6 months and young children aged 6.1 months – 48 months, which consisted of 3 sections: Sect. 1 contained questions on demographics characteristics including age, gestational age, gender, birth order, mode of delivery, prenatal complications, anthropometry, growth pattern, feeding practices, as well as medical history; Sect. 2 covered subject’s FGIDs history with explicit questions on gastrointestinal symptoms including stool pattern (using the validated Amsterdam and Bristol stool chart [21, 22]), spitting and crying habits for infants and young children; Sect. 3 collected information on the family social and economic details such as income, educational level of both parents and the occurrence of domestic violence in the household. The last section also included questions on adverse life events faced by the child and family, such as exposure of child or mother to physical or verbal abuse. Section 1 and 2 were collected via an interview from the research team while Sect. 3 was self-administered. After completing the questionnaire, the subjects were diagnosed using the ROME IV criteria and grouped according to the case definitions for infantile colic, regurgitation, dyschezia, functional diarrhoea and functional constipation [1, 3].

### Sample size calculation

The sample size was calculated using an estimated 10% prevalence for infantile colic and childhood constipation using Epi Info Stat Calc [4]. Assuming a confidence level of 95%, power of 80% and a precision found to the nearest 2%, a sample size of 750 infants in ages 0–6 months (group 1) and 750 young children in ages 6.1 months – 48 months (group 2) were considered adequate. Group 2 was further stratified for age according to the following categories: 6.1 – 12 months, 12.1 – 24 months, 24.1 – 36 months and 36.1 – 48 months (Table 1).

**Table 1:** Age stratification and the distribution of subjects

Age	Number of subjects according to age stratification	Number of subjects included for the final analysis
0—6 months	750	752
6.1—12 months	190	190
12.1—24 months	185	190
24.1—36 months	185	189
36.1—48 months	190	190
**Total subjects**	1500	1511

### Statistical analysis

The data was entered into a Microsoft Excel (Microsoft Office 365 MSO,16.0.012527.21230). The SPSS statistical analysis software (windows version 22.0) was used for the data analysis. Descriptive statistics were used to present the demographic characteristics such as age and weight of the sample and the prevalence of FGIDs in infants and young children. Descriptive statistics used includes means, medians, standard deviations, ranges, and percentages, as required by the distribution characteristics of each variable. Qualitative variables were expressed in terms of frequency and percentage. Quantitative variables were presented as means ± standard deviation (standard distribution) and median or quartile (non-standard distribution). The difference between quantitative variables was investigated by Student’s t-test. The difference between qualitative variables was compared by chi-square test (or McNemar). Multivariate logistic regression was used to evaluate the potential risk factors (such as demographic data, exposure to adverse events, social and economic factors) associated with the development of FGIDs. Odds ratio (OR) estimates and 95% confidence interval of OR, as well as the p-values of the chi-square test for each risk factor were provided. All statistical tests used a significance level of 0.05. Multiple logistic regression was performed on variables that were found to have significant associations.

### Ethical approval

The protocol, information letter to the parents/legal guardian and written informed consent form were approved by the Research Ethics Committee of the Vietnam National Children’s Hospital. The survey was conducted in accordance with the guidelines of the Declaration of Helsinki, the International Conference on Harmonization (ICH) guidelines on Good Clinical Practice (GCP), and applicable regulatory requirements of the Scientific and Medical Council National Children’s Hospital, Hanoi Medical University and Authority of Quynh Loi Kindergarten. The study was registered in the Netherlands Trial Registry (identifier: NL7286/NTR7495).

## Results

### Sample characteristics

Mothers of 1662 infants and young children between the age of 0 – 48 months old were invited to participate in the study. Of these, 1511 mothers (752 in group 1 aged 0–6 months and 759 in group 2 aged 6.1 – 48 months) completed the questionnaires for the final analysis (Table 1). Data was excluded from subjects who did not complete the screening procedure (*n* = 151).

All subjects recruited were Vietnamese, with a mean age of 3.17 ± 1.73 months for group 1 and 24.40 ± 12.99 months for group 2. A total of 829 subjects (54.9%) were males. Most subjects were living in the urban areas (78.2%). Around half of the subjects (51.2%) provided information on the range of income and most of the families (39.0%) had an annual household income below 273,000,000 VND (estimate around US$11,800). High perception of financial sufficiency was reported in 88.5% of the subjects stating that income met their needs. In terms of parental educational background, the majority of mothers (39.6%) had a Bachelor’s degree whereas the majority of the fathers (41.3%) had a high school education.

### Prevalence of FGIDs in infants and young children

In total, 151 subjects (10.0%) were diagnosed with at least one FGID, according to the Rome IV criteria. Only nine infants (0.6%) had more than one FGID of which the most common combination was infant colic and regurgitation. The prevalence of FGIDs among infants and young children according to residence area and age group is presented in Table 2. Infant regurgitation was the most prevalent FGID in early infancy (9.3%). The peak onset for regurgitation was observed between 2—3 months of age. Functional constipation was the most common disorder in the 7 months – 48 months old (5.6%). The prevalence of functional constipation was relatively high between the period of 5—6 months and 24—30 months of age. The overall prevalence for male subjects (3.9%) with reported functional constipation was significantly higher (*p* = 0.003) compared to female subjects (2.1%). All subjects at 0—6 months of age with functional constipation had reported formed stool consistency as evaluated by the Amsterdam Stool Chart. For the constipated subjects 6 months and above, stool consistencies of very hard with separate hard lumps, hard, and dry and lumpy were reported according to the Bristol stool chart. Colic prevalence of 2.5% was observed in early infancy, which peaked between the period of 1—2 months. Infant dyschezia was only reported in early infancy (0.9%), with peak onset between 2—3 months. Functional diarrhoea was not reported in both infants and young children.

**Table 2:** Prevalence of self-reported FGIDs according to residence area and age group

FGIDs	Age groups	Total	Prevalence	Rural	Urban	*p*-value	Male	Female	*p*-value
		(N)	n (%)	n/N (%)	n/N (%)		n/N (%)	n/N (%)	
Infant colic	0—6 months	752	19 (2.5%)	11/329 (3.3%)	8/423 (1.9%)	0.210	10/413 (2.4%)	9/339 (2.7%)	0.864
Infant regurgitation	3 weeks—6 months	685	64 (9.3%)	29/302 (9.6%)	58/573 (10.1%)	0.991	49/473 (10.4%)	38/402 (9.5%)	0.967
	7—12 months	190	23 (12.1%)						
Infant dyschezia	0—6 months	752	7 (0.9%)	3/329 (0.9%)	4/507 (0.8%)	0.490	4/461 (0.9%)	3/375 (0.8%)	0.400
	7—9 months	84	0 (0.0%)						
Functional diarrhoea	7—12 months	190	0 (0.0%)	0/0 (0.0%)	0/759 (0.0%)	-	0/416 (0.0%)	0/343 (0.0%)	-
	12.1—48 months	569	0 (0.0%)						
Functional constipation	0—6 months	752	14 (1.9%)	9/329 (2.7%)	37/1182 (3.1%)	0.114	32/829 (3.9%)	14/682 (2.1%)	0.003*
	7—12 months	190	0 (0.0%)						
	12.1—48 months	569	32 (5.6%)						

^*^Student’s t-test *p* < 0.05

### Association between FGIDs and early life factors

Supplementary table S1 shows early life factors and infant feeding practices against FGIDs outcome [see Supplementary file 2]. No association was found for infantile colic and dyschezia against gender, gestational age, birth weight, growth curve, mode of delivery and feeding. The risk of having infant regurgitation was significantly lower among infants with exclusive breastfeeding at 2 – 3 months (OR = 0.137, 95%CI = 0.028 – 0.675, *p* = 0.015), exclusive breastfeeding at 3 – 4 months (OR = 0.121, 95%CI = 0.028 – 0.516, *p* = 0.004) and formula initiation at 0 – 1 months (OR = 0.060, 95%CI = 0.010 – 0.378, *p* = 0.003). Being a male (OR = 3.665, 95%CI = 1.563 – 8.593, *p* = 0.003) and formula feeding initiation at 1 – 2 months (OR = 18.558, 95%CI = 1.569 – 219.444, *p* = 0.020) increased the risk of functional constipation.

With regards to feeding practices among infants 0 – 6 months old, a high percentage (95.2%) of breastfeeding initiation at birth was observed. The average duration of exclusive breastfeeding lasted up to 1.9 months, with the average starting age for formula feeding at 0.5 months. 71.8% of subjects between the age of 7 – 48 months reported the use of follow-on formula. In addition, 9.4% of the young children consumed additional vitamin and/or mineral supplements i.e. vitamin D, multi-vitamin, calcium and docosahexaenoic acid (DHA).

### Association between FGIDs and socio-demographic characteristics

Supplementary table S2 shows the association between FGIDs and socio-demographic factors including residence area, household income, family size, birth order, and parental education level [see Supplementary file 3]. No risk factor was identified for infantile colic and dyschezia. Higher paternal education level (Master’s degree) was associated significantly with a reduced risk of infant regurgitation (OR = 0.022, 95%CI = 0.002 – 0.327, *p* = 0.006). The risk of functional constipation was significantly higher for children in family with annual household income between 273,000,000 – 546,999,999 VND or estimate around 11,800 – 23,800 USD (OR = 5.887, 95%CI = 1.794 – 19.321, *p* = 0.003) and family with one child only (OR =  > 999.999, 95%CI =  > 999.999—> 999.999, *p* < 0.001).

### Association between FGIDs and stressful life events

Supplementary table S3 shows the association between FGIDs and stressful events as faced by the main caregiver and the child [see Supplementary file 4]. Most of the subjects reported that neither they nor their children were exposed to any of the listed stressful events.

## Discussion

This study unveiled the prevalence and risk factors of FGIDs in Vietnamese infants and young children for the first time using the Rome IV criteria. We observed around 10% of infants and young children from 0 to 48 months are suffering from at least one FGID. Infantile regurgitation (9.3%) was the most prevalent FGID among Vietnamese infants below 6 months of age. The other FGIDs (colic, functional diarrhoea, and infantile dyschezia) have low prevalence between 0- 2.5%. The prevalence of infantile regurgitation increased to 12.1% for infants 7 – 12 months old. For infants and young children 7 months – 48 months old, functional constipation (5.6%) was the most common disorder.

Currently, Vietnam has a population of more than 95 million, of which about 25% are children under the age of 15 years. As a developing country, Vietnam is experiencing the challenges of globalisation that leads to rapid change in socioeconomic status and their cultural foundations especially in the health care services. In this study we found a relatively low prevalence of FGIDs in Vietnamese infants and young children. Previous studies employing Rome IV criteria in the assessment of paediatric FGIDs reported 24.7% in the United States [5], over 30% in the African countries [7], 24.7% in Europe [23], 27.3% in China [14], and 14.6% in Malaysia [15]. The prevalence of FGIDs in Vietnam appeared to be akin to the findings in a study conducted in Malaysia. Of the 534 healthy infants recruited in Malaysia, infantile regurgitation was also found to be the most prevalent FGID (10.5%), while the other FGIDs diagnoses have low prevalence between 1 – 2% [15]. The observed similarity could be attributed to the commonality of urban setting in Southeast Asia. Up to 78.2% of Vietnamese subjects in our study were living in the urban areas, whereas the Malaysia study was conducted in a well-baby clinic located in a large urban hospital [15].

Infantile regurgitation could be considered as the most common FGID among infants and young children [5, 23–25]. Our current study observed infant regurgitation at a prevalence rate of 9.3% in the 0 – 6 months age group, which increased to 12.1% in the 7 – 12 months age group. The peak onset of regurgitation was observed between 2 – 3 months of age. Comparing our results with the American (24.1%), African (39.7%), European (13.8%), Chinese (33.9%), and Malaysian (10.5%) studies which also used the Rome IV criteria, the prevalence of infantile regurgitation in the current study was closer to the Malaysian study [5, 7, 14, 15, 23]. Contradictorily, the increasing prevalence of infantile regurgitation in the older infants is conflicting with previous findings [5, 14, 23]. We observed that majority of the subjects at 0 – 6 months old (53.6%) consumed breastmilk only. For older infants at 7 – 12 months old, the breastfeeding only rate reduced to 1.6%, with 43.7% consumed breastmilk and additional foods, 26.8% consumed breastmilk and formula, and 21.1% consumed breastmilk, formula and additional foods. It is plausible that the introduction of formula and weaning foods could lead to overfeeding. Overfeeding exacerbates infantile regurgitation while conservative measures such as upright positioning after feeding, elevating the head of the bed, prone positioning (for infants over 6 months of age), and providing small and frequent feeds are often recommended for parental management [26]. Along with the maturity of the digestive tract, most infants usually outgrow the physiological reflux after one year of age [27].

Our study found that the mode of feeding early in life influences the risk of infant regurgitation. Specifically, the risk was significantly lower among infants with exclusively breastfeeding at 2 – 3 months, exclusively breastfeeding at 3 – 4 months and formula initiation at 0 – 1 months. In term of feeding practices among the Vietnamese infants 0 – 6 months old, 95.2% of caregivers initiated breastfeeding at birth, with the exclusive breastfeeding rate of 53.6%. Up to 44.5% of infants 0 – 6 months old consumed formula milk, with the average formula initiation at 0.5 month. Overfeeding could be a cause of infant regurgitation and in general, parents tend to overfeed while bottle feeding because they are likely less responsive to infants’ signals of hunger and satiation [26, 28]. Some studies suggested that breastfeeding lowers the risk of regurgitation [15, 23, 29], while others observed mixed findings [14] or no correlation [30]. Breast fed infants were observed to have a faster gastric emptying that can potentially contribute to a lower incidence of reflux [31, 32]. Furthermore, our study showed that higher paternal education level was associated with a reduced risk of infant regurgitation potentially due to higher parental awareness of correct feeding positioning.

Low prevalence was observed for the other FGIDs in Vietnamese infants with colic at 2.5% and infant dyschezia at 0.9%. Our study found no occurrence of functional diarrhoea in both infants and young children, similar to the observation in the US study [5]. The onset periods of several FGIDs were consistent as previously reported in China [14], i.e. colic (1 – 2 months), regurgitation (2 – 3 months), dyschezia (around 2 months), and functional constipation (two onsets around 5 months and 24—30 months). No association was found between the low prevalent FGIDs and the monitored socio-demographical aspects.

For Vietnamese young children between 7 – 48 months old, functional constipation was the most common FGID at the rate of 5.6%. During this window, the prevalence was relatively high between the period of 24—30 months of age. Our finding was in accordance with previous studies, that reported a higher prevalence of functional constipation in young children with a median age of onset of 27.6 months [24, 33, 34]. Our study showed that male gender, formula feeding initiation at 1 – 2 months, children in family with lower-middle household income per annum between 273,000,000 – 546,999,999 VND (or estimate around 11,800 – 23,800 USD), and family with one child only were significantly associated with an increased risk of functional constipation. In contrast, a recent systematic review and a meta-analysis reported no difference in the prevalence of functional constipation in older children [35]. However, the systematic review of epidemiology of FGIDs in infants and toddlers did not report any predisposing socio-demographic factors [4].

Stressful life events could predispose children to FGIDs. A study conducted among children of 6 – 48 months in Sri Lanka using ROME III criteria reported a higher prevalence of functional constipation in infants and young children subjected to violence and living with mothers exposed to violence [36]. Furthermore, functional constipation was found to be significantly associated with underweight, a sign of potential growth faltering. Therefore, we hypothesise that maternal and child exposure to violence may increase the risk of FGIDs in infants and young children. However, in our study, the majority of the Vietnamese subjects reported that neither they nor their children were exposed to any of the listed stressful life events. Besides, all the subjects recruited has a normal growth pattern of neither under- nor overweight. Our findings are compatible with a recent study from China which reported non-exposure of mothers and young children to violence [14].

Our study managed to capture a large sample size of the Vietnamese population. Besides, questionnaire was tested and validated in the local language with extra care taken to provide explanation to parents during the data collection process. However, this study has several limitations that should be acknowledged. Although we recruited a large sample size, the data was skewed towards urban setting. In addition, we used parental report to interpret child’s symptoms. Parents may not be aware of non-observable symptoms and it is subjected to the inherent reporting bias [5]. Besides, our cross-sectional design presented only a limited facet on the growth trajectory. For future research, longitudinal studies could be performed to assess the impact of FGIDs throughout childhood and ideally into the adulthood, which could provide a better understanding on the associated risk factors.

## Conclusions

This is the first study to determine the prevalence and risk factors of FGIDs in Vietnamese infants and young children using the Rome IV criteria. We found that 10.0% of Vietnamese infant and young children fulfilled the Rome IV criteria for at least one FGID. The prevalence of paediatric FGIDs in Vietnamese population was relatively low compared to the published literature. Our data indicated that the most common FGIDs in infants was regurgitation, while functional constipation was most prominent FGID among young children. Risk factors including gender, paternal education, household income, duration of exclusive breastfeeding and age of formula initiation were identified.

## Supplementary Information

**Additional file 1.** Questionnaire.

**Additional file 2: Table S1.** Early life factors and infant feeding practices against FGIDs.

**Additional file 3: Table S2.** Socio-demographic characteristics against FGIDs.

**Additional file 4: Table S3.** Stressful life events against FGIDs.

## Data Availability

Data generated or analyzed during this study are included in this published article [and its supplementary information files].

## Abbreviation

FGIDs: Functional Gastrointestinal Disorders
