# Supplementary material for: Prevalence and risk factors of functional gastrointestinal disorders in Vietnamese infants and young children
Source: BMC Pediatr. 2022 May 27;22:315. doi: 10.1186/s12887-022-03378-z (PMC9137065; doi:10.1186/s12887-022-03378-z)
Supplement: Supplementary file 2 — Additional file 2: Table S1. Early life factors and infant feeding practices against FGIDs. [file 12887_2022_3378_MOESM2_ESM.doc]

Supplementary table S1. Early life factors and infant feeding practices against FGIDs.

|  | | **Infant Colic** | | **Infant Regurgitation** | | **Infant Dyschezia** | | **Functional Diarrhoea** | | **Functional Constipation** | |
| --- | --- | --- | --- | --- | --- | --- | --- | --- | --- | --- | --- |
|  |  | **OR**  **(95% CI)** | **p value** | **OR**  **(95% CI)** | **p value** | **OR**  **(95% CI)** | **p value** | **OR**  **(95% CI)** | **p value** | **OR**  **(95% CI)** | **p value** |
| **Gender** | Male | 1.098  (0.378-3.186) | 0.864 | 0.520  (0.268-1.010) | 0.967 | 0.477  (0.085-2.667) | 0.400 | - | - | 3.665  (1.563-8.593) | **0.003**** |
|  | Female | MV | MV | MV | 0.967 | MV | MV | - | - | MV | MV |
| **Gestational Age (weeks)** | <34 | 0.856  (<0.001->999.999) | 0.971 | 0.315  (0.018-5.586) | 0.431 | 3.803  (<0.001->999.999) | 0.818 | - | - | <0.0013  (<0.001-C) | 0.993 |
|  | 35 – 37 | 0.653  (0.125-3.421) | 0.614 | 0.406  (0.144-1.146) | 0.089 | 1.735  (0.060-50.113) | 0.748 | - | - | 1.2032  (0.417-3.474) | 0.732 |
|  | 38 – 42 | MV | MV | MV | MV | MV | MV | - | - | MV | MV |
|  | >42 | - | - | - | - | - | - | - | - | - | - |
| **Birth Weight (kg)** | <1.5 | MV | MV | 0.539  (0.007-38.861) | 0.777 | 0.314  (<0.001->999.999) | 0.921 | - | - | 0.025  (<0.001-C) | 0.999 |
|  | 1.6 – 2.0 | 2.708  (0.001-7755.737) | 0.806 | 1.305  (0.047-36.387) | 0.875 | 0.430  (<0.001->999.999) | 0.889 | - | - | <0.001  (<0.001-C) | 0.992 |
|  | 2.1 – 2.5 | 1.635  (0.043-82.846) | 0.792 | 0.942  (0.165-5.383) | 0.946 | 1.298  (0.009-187.559) | 0.918 | - | - | 0.583  (0.094-3.625) | 0.563 |
|  | 2.6 – 3.0 | 1.337  (0.411-4.354) | 0.629 | 1.454  (0.690-3.067) | 0.325 | 0.689  (0.130-3.648) | 0.662 | - | - | 0.749  (0.322-1.742) | 0.502 |
|  | >3.0 | MV | MV | MV | MV | MV | MV | - | - | MV | MV |
| **Growth Curve** | Normal | - | - | - | - | - | - | - | - | - | - |
|  | Over-weight | - | - | - | - | - | - | - | - | - | - |
|  | Under-weight | - | - | - | - | - | - | - | - | - | - |
|  | Stunting | - | - | - | - | - | - | - | - | - | - |
|  | Wasted | - | - | - | - | - | - | - | - | - | - |
| **Mode of Delivery** | Vaginal | 0.558  (0.005-64.622) | 0.810 | <0.001  (<0.001->999.999) | 0.986 | 0.684  (<0.001-951.310) | 0.918 | - | - | >999.999  (<0.001-C) | 0.994 |
|  | Elective C-section | 0.617  (0.005-73.787) | 0.843 | <0.001  (<0.001->999.999) | 0.986 | 0.890  (0.001-1325.696) | 0.975 | - | - | >999.999  (<0.001-C) | 0.994 |
|  | Emergency C-section | MV | MV | MV | MV | MV | MV | - | - | MV | MV |
|  | Forceps Delivery | - | - | - | - | - | - | - | - | - | **-** |
| **Exclusively Breast-feeding Duration (months)** | <1 | 1.078  (0.031-37.383) | 0.967 | 5.838  (0.730-46.663) | 0.096 | 2.472  (0.024-253.370) | 0.702 | - | - | 2.237  (0.004->999.999) | 0.801 |
|  | 1 – 2 | 0.675  (0.032-14.033) | 0.799 | 0.427  (0.065-2.813) | 0.376 | 0.668  (0.014-31.388) | 0.837 | - | - | 0.653  (0.061-6.942) | 0.724 |
|  | 2 – 3 | 0.156  (0.015-1.566) | 0.114 | 0.137  (0.028-0.675) | **0.015*** | 0.584  (0.018-19.126) | 0.762 | - | - | 0.972  (0.118-7.989) | 0.979 |
|  | 3 – 4 | 0.483  (0.045-5.202) | 0.548 | 0.121  (0.028-0.516) | **0.004**** | 0.478  (0.019-12.078) | 0.654 | - | - | 0.169  (0.007-3.838) | 0.264 |
|  | 4 – 6 | MV | MV | MV | MV | MV | MV | - | - | MV | MV |
| **Age of Formula Feeding Initiation (months)-** | 0 – 1 | 0.616  (0.044-8.638) | 0.719 | 0.060  (0.010-0.378) | **0.003**** | 0.514  (0.017-15.247) | 0.700 | - | - | 0.984  (0.002-446.912) | 0.996 |
|  | 1 – 2 | 1.413  （0.001-1945.690） | 0.925 | >999.999  (<0.001->999.999) | 0.990 | 1.311  (<0.001->999.999) | 0.958 | - | - | 18.558  (1.569-219.444) | **0.020*** |
|  | 2 – 3 | 4.435  （0.024-818.750） | 0.576 | 2.784  (0.216-35.917) | 0.433 | 1.493  (0.001-2881.382) | 0.917 | - | - | 7.024  (0.799-61.748) | 0.079 |
|  | 3 – 4 | 2.433  (0.015-401.887) | 0.733 | 1.969  (0.192-20.140) | 0.568 | 2.584  (0.004-1781.583) | 0.776 | - | - | 3.706  (0.323-42.457) | 0.292 |
|  | 4 – 5 | 0.989  (0.001-842.193) | 0.997 | 0.557  (0.012-26.130) | 0.765 | 1.311  (<0.001->999.999) | 0.959 | - | - | 2.114  (0.088-50.586) | 0.644 |
|  | 5 – 6 | 1.017  (<0.001-2531.842) | 0.997 | >999.999  (<0.001->999.999) | 0.984 | 1.043  (0.001-914.078) | 0.990 | - | - | <0.001  (<0.001-C) | 0.990 |
|  | >6 | - | - | - | - | - | - | - | - | 0.423  (0.073-2.444) | 0.336 |
|  | Never | MV | MV | MV | MV | MV | MV | - | - | MV | MV |

*: p < 0.05, **: p < 0.01, -: no OR (95% CI) and p value, C: Floating point overflow occurred while computing this statistic. Its value is therefore set to system missing, MV: missing value
