# Supplementary material for: Prevalence and risk factors of functional gastrointestinal disorders in Vietnamese infants and young children
Source: BMC Pediatr. 2022 May 27;22:315. doi: 10.1186/s12887-022-03378-z (PMC9137065; doi:10.1186/s12887-022-03378-z)
Supplement: Supplementary file 3 — Additional file 3: Table S2. Socio-demographic characteristics against FGIDs. [file 12887_2022_3378_MOESM3_ESM.doc]

Supplementary table S2. Socio-demographic characteristics against FGIDs.

|  | | **Infant Colic** | | **Infant Regurgitation** | | **Infant Dyschezia** | | **Functional Diarrhoea** | | **Functional Constipation** | |
| --- | --- | --- | --- | --- | --- | --- | --- | --- | --- | --- | --- |
|  |  | **OR**  **(95% CI)** | **p value** | **OR**  **(95% CI)** | **p value** | **OR**  **(95% CI)** | **p value** | **OR**  **(95% CI)** | **p value** | **OR**  **(95% CI)** | **p value** |
| **Residence Area** | Urban | 2.109  (0.656-6.785) | 0.210 | 1.177  (0.565-2.454) | 0.991 | 1.821  (0.332-9.989) | 0.490 | - | - | 0.392  (0.123-1.251) | 0.114 |
|  | Rural | MV | MV | MV | 0.991 | MV | MV | - | - | MV | MV |
| **Annual Household Income (VND)** | <273,000,000 | 4.020  (<0.001->999.999) | 0.878 | <0.001  (<0.001->999.999) | 0.941 | 0.521  (<0.001->999.999) | 0.964 | - | - | 1.050  (0.388-2.841) | 0.924 |
|  | 273,000,000 – 546,999,999 | 1.890  (<0.001->999.999) | 0.944 | <0.001  (<0.001->999.999) | 0.391 | 0.700  (<0.001->999.999) | 0.980 | - | - | 5.887  (1.794-19.321) | **0.003**** |
|  | *547,000,000 – 955,999,999* | MV | MV | MV | MV | MV | MV | - | - | <0.001  (<0.001-<0.001) | MV |
|  | *956,000,000 – 1,364,999,999* | - | - | - | - | - | - | - | - | - | - |
|  | 1,365,000,000 – 2,722,999,999 | - | - | - | - | - | - | - | - | - | - |
|  | >2,723,000,000 | - | - | - | - | - | - | - | - | - | - |
|  | No answer | MV | MV | MV | MV | MV | MV | - | - | MV | MV |
| **Family Size** | 1 child only | 6.192  (<0.001->999.999) | 0.876 | <0.001  (<0.001->999.999) | 0.995 | 0.227  (<0.001->999.999) | 0.931 | - | - | >999.999  (>999.999->999.999) | **<0.001***** |
|  | 2 – 3 Children | 5.279  (<0.001->999.999) | 0.878 | <0.001  (<0.001->999.999) | 0.996 | 0.139  (<0.001->999.999) | 0.902 | - | - | >999.999  (>999.999->999.999) | MV |
|  | ≥4 Children | MV | MV | MV | MV | MV | MV | - | - | MV | MV |
| **Birth Order** | 1 | 0.386  (<0.001->999.999) | 0.831 | 12.010  (0.005->999.999) | 0.527 | 1.470  (<0.001->999.999) | 0.952 | - | - | 1.238  (0.274-6.011) | 0.752 |
|  | 2 | 0.572  (0.096-3.403) | 0.539 | 1.847  (0.770-4.426) | 0.169 | 2.343  (0.356-15.401) | 0.376 | - | - | 0.689  (0.248-1.919) | 0.476 |
|  | 3 | MV | MV | MV | MV | MV | MV | - | - | MV | MV |
|  | 4 | 0.034  (<0.001->999.999) | 0.756 | 0.248  (<0.001->999.999) | 1.000 | 0.105  (<0.001->999.999) | 0.893 | - | - | 7.361  (<0.001-C) | 0.999 |
|  | 5 | MV | MV | MV | MV | MV | MV | - | - | MV | MV |
| **Paternal Education Level** | Primary | 12.636  (<0.001->999.999) | 0.691 | >999.999  (<0.001->999.999) | 0.985 | 14.871  (<0.001->999.999) | 0.735 | - | - | <0.001  (<0.001-C) | 0.991 |
|  | Secondary | 1.984  (0.114-34.393) | 0.638 | 0.813  (0.147-4.492) | 0.813 | 8.486  (0.117-617.146) | 0.328 | - | - | 1.757  (0.269-11.470) | 0.556 |
|  | High School | 1.490  (0.175-12.710) | 0.761 | 0.982  (0.234-4.126) | 0.980 | 2.375  (0.237-23.811) | 0.462 | - | - | 1.277  (0.297-5.493) | 0.743 |
|  | College | MV | MV | MV | MV | MV | MV | - | - | MV | MV |
|  | Bachelors | 0.971  (0.107-8.773) | 0.979 | 0.639  (0.143-2.848) | 0.557 | 3.230  (0.250-41.768) | 0.369 | - | - | 0.397  (0.080-1.969) | 0.258 |
|  | Masters | 1.795  (0.008-409.249) | 0.833 | 0.022  (0.002-0.327) | **0.006**** | 3.832  (<0.001->999.999) | 0.788 | - | - | <0.001  (<0.001-C) | 0.995 |
|  | PhD | - | - | - | - | - | - | - | - | - | - |
| **Maternal Education Level** | Primary | 2.237  (<0.001->999.999) | 0.893 | 0.167  (0.010-2.771) | 0.212 | 0.701  (<0.001->999.999) | 0.960 | - | - | 4.274  (0.285-64.158) | 0.293 |
|  | Secondary | 0.503  (0.056-4.491) | 0.539 | 0.810  (0.206-3.187) | 0.763 | 0.620  (0.040-9.535) | 0.732 | - | - | 2.654  (0.479-14.703) | 0.264 |
|  | High School | 1.251  (0.207-7.577) | 0.807 | 0.959  (0.332-2.765) | 0.938 | 2.538  (0.213-30.204) | 0.461 | - | - | 1.046  (0.247-4.425) | 0.951 |
|  | College | MV | MV | MV | MV | MV | MV | - | - | MV | MV |
|  | Bachelors | 1.067  (0.211-5.403) | 0.937 | 1.683  (0.585-4.845) | 0.334 | 0.854  (0.091-8.020) | 0.890 | - | - | 1.722  (0.395-7.502) | 0.469 |
|  | Masters | 0.135  (0.011-1.673) | 0.119 | 0.230  (0.034-1.551) | 0.131 | 0.987  (<0.001->999.999) | 0.998 | - | - | <0.002  (<0.001-C) | 0.996 |
|  | PhD | - | - | - | - | - | - | - | - | - | - |

*: p < 0.05, **: p < 0.01, ***: p < 0.001, -: no OR (95% CI) and p value, C: Floating point overflow occurred while computing this statistic. Its value is therefore set to system missing, MV: missing value
