# Supplementary material for: Prevalence and risk factors of functional gastrointestinal disorders in Vietnamese infants and young children
Source: BMC Pediatr. 2022 May 27;22:315. doi: 10.1186/s12887-022-03378-z (PMC9137065; doi:10.1186/s12887-022-03378-z)
Supplement: Supplementary file 4 — Additional file 4: Table S3. Stressful life events against FGIDs. [file 12887_2022_3378_MOESM4_ESM.doc]

Supplementary table S3. Stressful life events against FGIDs.

|  | | **Infant Colic** | | **Infant Regurgitation** | | **Infant Dyschezia** | | **Functional Diarrhoea** | | **Functional Constipation** | |
| --- | --- | --- | --- | --- | --- | --- | --- | --- | --- | --- | --- |
|  |  | **OR**  **(95% CI)** | **p value** | **OR**  **(95% CI)** | **p value** | **OR**  **(95% CI)** | **p value** | **OR**  **(95% CI)** | **p value** | **OR**  **(95% CI)** | **p value** |
| **Income Meets Needs** | Yes | 1.047  (0.198-5.541) | 0.956 | 2.330  (0.751-7.230) | 0.143 | 0.336  (0.060-1.868) | 0.213 | - | - | MV | MV |
|  | No | MV | MV | MV | MV | MV | MV | - | - | 1.424  (0.535-3.790) | 0.479 |
| **Family Relationship** | Very Good | 1.717  (<0.001->999.999) | 0.969 | <0.001  (<0.001->999.999) | 0.999 | >999.999  (>999.999->999.999) | **0.005**** | - | - | >999.999  (<0.001-C) | 0.999 |
|  | Good | 1.411  (<0.001->999.999) | 0.980 | <0.001  (<0.001->999.999) | 0.999 | >999.999  (>999.999->999.999) | **0.005**** | - | - | >999.999  (<0.001-C) | 0.999 |
|  | Bad | 3.057  (<0.001->999.999) | 0.936 | >999.999  (<0.001->999.999) | 0.995 | 0.065  (<0.001->999.999) | 0.902 | - | - | >999.999  (<0.001-C) | 0.999 |
|  | Very Bad | MV | MV | MV | MV | MV | MV | - | - | MV | MV |
| **Verbal Violence on Interviewee (mother / caregiver)** | Never | 3.022  (<0.001->999.999) | 0.902 | >999.999  (<0.001->999.999) | 0.998 | 0.343  (<0.001->999.999) | 0.942 | - | - | 0.101  (<0.001-C) | 0.999 |
|  | Once in 2 months | 19.270  (<0.001->999.999) | 0.770 | >999.999  (<0.001->999.999) | 0.998 | 0.3365  (<0.001->999.999) | 0.952 | - | - | 0.014  (<0.001-C) | 0.999 |
|  | Once a month | MV | MV | >999.999  (<0.001->999.999) | 0.998 | 0.176  (<0.001->999.999) | 0.917 | - | - | 0.081  (<0.001-C) | 0.999 |
|  | >Once a month | - | - | >999.999  (<0.001->999.999) | 0.999 | MV | MV | - | - | 0.151  (<0.001-C) | 0.999 |
|  | Once a week | MV | MV | MV | MV | MV | MV | - | - | 0.736  (<0.001-C) | 1.000 |
|  | >Once a week | MV | MV | MV | MV | MV | MV | - | - | 0.723  (<0.001-C) | 1.000 |
|  | Daily | MV | MV | MV | MV | MV | MV | - | - | MV | MV |
| **Physical Violence on Interviewee (mother / caregiver)** | Never | MV | MV | 26437.821  (26437.821-26437.821) | MV | MV | MV | - | - | 20.126  (<0.001-C) | 0.999 |
|  | Once in 2 months | MV | MV | >999.999  (<0.001->999.999) | 0.989 | MV | MV | - | - | 20.635  (<0.001-C) | 0.999 |
|  | Once a month | MV | MV | MV | MV | MV | MV | - | - | <0.001  (MV-C) | 0.998 |
|  | >Once a month | - | - | MV | MV | MV | MV | - | - | MV | MV |
|  | Once a week | - | - | - | - | - | - | - | - | - | - |
|  | >Once a week | - | - | - | - | - | - | - | - | - | - |
|  | Daily | - | - | - | - | - | - | - | - | - | - |
| **Verbal Violence on Subject (Infant / young child)** | Never | MV | MV | MV | MV | MV | MV | - | - | 1.016  (0.003-377.554) | 0.996 |
|  | Once in 2 months | - | - | - | - | - | - | - | - | 3.436  (0.006->999.999) | 0.707 |
|  | Once a month | - | - | - | - | - | - | - | - | 2.041  (0.005-760.846) | 0.813 |
|  | >Once a month | - | - | - | - | - | - | - | - | 23.689  (0.054->999.999) | 0.308 |
|  | Once a week | - | - | - | - | - | - | - | - | 5.752  (0.014->999.999) | 0.568 |
|  | >Once a week | - | - | - | - | - | - | - | - | 5.374  (0.015->999.999) | 0.573 |
|  | Daily | - | - | - | - | - | - | - | - | MV | MV |
| **Physical Violence on Subject (Infant / young child)** | Never | MV | MV | MV | MV | MV | MV | - | - | >999.999  (<0.001-C) | 0.997 |
|  | Once in 2 months | - | - | - | - | - | - | - | - | >999.999  (<0.001-C) | 0.997 |
|  | Once a month | - | - | - | - | - | - | - | - | >000.999  (<0.001-C) | 0.997 |
|  | >Once a month | - | - | - | - | - | - | - | - | >999.999  (<0.001-C) | 0.996 |
|  | Once a week | - | - | - | - | - | - | - | - | >999.999  (<0.001-C) | 0.996 |
|  | >Once a week | - | - | - | - | - | - | - | - | MV | MV |
|  | Daily | - | - | - | - | - | - | - | - | - | - |

*: p < 0.05, **: p < 0.01, -: no OR (95% CI) and p value, C: Floating point overflow occurred while computing this statistic. Its value is therefore set to system missing, MV: missing value
